# NUTRITIONAL OUTCOMES AFTER ONE ANASTOMOSIS GASTRIC BYPASS VERSUS ROUX-EN-Y GASTRIC BYPASS: A ONE-YEAR COMPARATIVE STUDY

**DOI:** 10.1590/S0004-2803.24612026-040

**Published:** 2026-10-30

**Authors:** Priscilla SAMPAIO, Denis PAJECKI, Marco Aurelio SANTO, Maria Carolina Gonçalves DIAS, Marco Aurelio SANTO

**Affiliations:** 1 Universidade de São Paulo, Faculdade de Medicina, Hospital das Clínicas HCFMUSP, Divisão de Nutrição e Dietética, São Paulo, SP, Brasil.; 2 Universidade de São Paulo, Faculdade de Medicina, Hospital das Clínicas HCFMUSP, Departamento de Gastroenterologia, Disciplina de Cirurgia do Aparelho Digestivo e Coloproctologia, Unidade de Cirurgia Bariátrica e Metabólica, São Paulo, SP, Brasil.

**Keywords:** Bariatric surgery, one anastomosis gastric bypass, Roux-en-Y gastric bypass, nutritional status, gastrointestinal symptoms, food tolerance, Cirurgia bariátrica e metabólica, derivação gastrojejunal com anastomose única, derivação gástrica em Y-de-Roux, estado nutricional, sintomas gastrointestinais, tolerância alimentar

## Abstract

**Background:**

Metabolic and bariatric surgery is an effective obesity treatment. One anastomosis gastric bypass (OAGB) has emerged as an alternative to Roux-en-Y gastric bypass (RYGB); however, evidence regarding its postoperative nutritional outcomes remains limited. Objective: To compare food tolerance, gastrointestinal symptoms, and nutritional status between OAGB and RYGB one year after surgery.

**Methods:**

This retrospective, observational, comparative study included 46 patients (16 OAGB and 30 RYGB). Weight loss, laboratory parameters, dietary intake, food tolerance, and gastrointestinal symptoms were evaluated one year postoperatively. Linear regression models were adjusted for relevant confounding variables, including age, sex, and baseline values when appropriate.

**Results:**

The percentage of total weight loss (29.55% vs 26.32%) and excess weight loss (63.36% vs 58.29%) were similar between RYGB and OAGB groups (*P*>0.05). A high prevalence of vitamin D deficiency was observed in both groups (RYGB: 82% vs. OAGB: 92%). Elevated parathyroid hormone levels were more frequent in the OAGB group (75% vs 50%), although the difference was not statistically significant. OAGB patients showed numerically lower serum iron and reduced transferrin saturation; however, none of the patients developed anemia. Mean protein intake met the recommended levels in both groups, whereas fiber intake was below the minimum recommended level. OAGB patients presented significantly lower gastrointestinal symptom scores for diarrhea, indigestion, and total score (*P*<0.05). Food tolerance was satisfactory and similar between groups.

**Conclusion:**

Under structured multidisciplinary follow-up, OAGB and RYGB demonstrated comparable nutritional outcomes. OAGB appears to be a nutritionally safe alternative at one year of follow-up, with the need for ongoing monitoring of iron and vitamin D status.

## HIGHLIGHTS

• OAGB and RYGB achieved comparable weight loss, dietary intake, and food tolerance. OAGB showed lower gastrointestinal symptom scores.

• Vitamin D deficiency was highly prevalent in both groups; elevated PTH and subclinical iron metabolism alterations were more frequent in the OAGB group.

• No clinically relevant food intolerance was observed with either surgical technique.

• Under structured multidisciplinary follow-up, OAGB is a nutritionally safe alternative to RYGB, requiring ongoing monitoring of iron and vitamin D status.

## RESUMO

**Contexto:**

A cirurgia bariátrica e metabólica é uma estratégia eficaz no tratamento da obesidade. A derivação gastrojejunal com anastomose única (OAGB) tem sido proposta como alternativa à derivação gástrica em Y-de-Roux (DGYR), no entanto, as evidências sobre seus desfechos nutricionais pós-operatórios ainda são limitadas.

**Objetivo:**

Comparar a tolerância alimentar, os sintomas gastrointestinais e o estado nutricional entre pacientes submetidos à OAGB e à DGYR um ano após a cirurgia.

**Métodos:**

Estudo observacional, retrospectivo e comparativo, envolvendo 46 pacientes (16 OAGB e 30 DGYR). Foram avaliados perda de peso, exames laboratoriais, consumo alimentar, tolerância alimentar e sintomas gastrointestinais um ano após o procedimento. Modelos de regressão linear foram ajustados para variáveis de confusão relevantes, incluindo idade, sexo e valores basais quando apropriado.

**Resultados:**

O percentual de perda de peso total (29,55% vs 26,32%) e de perda do excesso de peso (63,36% vs 58,29%) foram semelhantes entre os grupos DGYR e OAGB (*P*>0,05). Alta prevalência de deficiência de vitamina D foi observada em ambos os grupos (DGYR: 82% vs OAGB: 92%). Níveis elevados de paratormônio foram mais frequentes no grupo OAGB (75% vs 50%), embora sem diferença estatisticamente significante. O grupo OAGB apresentou menores níveis de ferro sérico e saturação de transferrina; no entanto, nenhum paciente desenvolveu anemia. A ingestão proteica média atendeu às recomendações em ambos os grupos, enquanto o consumo de fibras ficou abaixo do mínimo recomendado. Pacientes submetidos à OAGB apresentaram escores significativamente menores de sintomas gastrointestinais para diarreia, indigestão e escore total (*P*<0,05). A tolerância alimentar foi satisfatória e semelhante entre os grupos.

**Conclusão:**

Sob acompanhamento multidisciplinar estruturado, OAGB e DGYR demonstraram desfechos nutricionais comparáveis. A OAGB parece ser uma alternativa nutricionalmente segura no primeiro ano de seguimento, com necessidade de monitoramento contínuo do estado de ferro e vitamina D.

## INTRODUCTION

Obesity is a chronic metabolic disease and a major global public health concern, with a rise in prevalence over recent decades^1^ ^,^ ^2^. Bariatric and metabolic surgery remains the most effective, long-lasting treatment for obesity, contributing to substantial weight loss, remission of obesity-related comorbidities, improvement in quality of life, and survival^3^ ^,^ ^4^.

Among available bariatric and metabolic proce­dures, Roux-en-Y gastric bypass (RYGB) has tradi­tionally been considered one of the most effective options for obesity treatment, remaining the most performed bariatric and metabolic surgery in Brazil and the second most performed worldwide^5^ ^,^ ^6^. However, the heterogeneity among patients with obesity has driven the need for individualized care and the search for alternative surgical techniques^7^ ^,^ ^8^. In this context, one anastomosis gastric bypass (OAGB) has emerged as a promising bariatric and metabolic surgical technique. This technique involves the creation of a long gastric pouch with a single gastrojejunal anastomosis, approximately 200 cm to the ligament of Treitz^9^. Initially described in 2001 by Rutledge et al.^9^, OAGB has gained increasing attention and currently ranks as the third most frequently performed bariatric procedure worldwide^5^.

Despite the growing global popularity of OAGB, its relatively recent implementation leaves gaps in scientific literature, particularly in nutritional effects. Both RYGB and OAGB are restrictive and malabsorptive procedures that may predispose patients to nutritional deficiencies, food intolerance, and gastrointestinal consequences^10^ ^,^ ^11^. Therefore, this study aimed to compare food tolerance, gastrointestinal symptoms, and nutritional status between patients undergoing OAGB or RYGB one year after surgery.

## METHODS

This observational, comparative, retrospective study included patients with obesity who underwent either one-anastomosis gastric bypass (OAGB) or Roux-en-Y gastric bypass (RYGB). Patients were evaluated with at least one year of postoperative follow-up.

In RYGB, a gastric pouch (~30 to 40 mL), without a silicon ring, was created along the lesser curvature, followed by a Roux-en-Y reconstruction with a 100 cm biliopancreatic limb and a 100 cm alimentary limb. In OAGB, a long narrow gastric pouch (~150ml) was created along the lesser curvature with a single gastrojejunal anastomosis 200 cm distal to the ligament of Treitz.

Eligible participants had a body mass index (BMI) ≥35 kg/m² and were aged between 35 and 65 years. Surgical procedures were performed between August 2021 and December 2024 at the Bariatric and Metabolic Surgery Service of Hospital das Clínicas, University of São Paulo Medical School (HCFMUSP), Brazil, a public tertiary referral hospital. Exclusion criteria were patients with previous bariatric or gastric surgery, personal or family history of gastric or esophageal cancer, chronic atrophic gastritis or other conditions associated with increased gastric malignancy risk, and previous diagnosis of gastroesophageal reflux disease (GERD) or Barrett’s esophagus. All patients received standardized postoperative nutritional counseling and micronutrient supplementation according to institutional protocols^12^ ^,^ ^13^.

The groups were matched according to BMI, sex, and age in an approximate 1:2 ratio. The sample was defined by convenience according to the institutional operational capacity of the service. The study protocol was approved by Research Ethics Committee of HCFMUSP. All participants signed an informed consent prior to their inclusion.

Anthropometric data (last preoperative, maximal preoperative, and one-year postoperative weights and BMIs), total weight loss (TWL), and excess weight loss (EWL) were obtained from institutional electronic medical records. For EWL, it was considered the ideal weight corresponding to a BMI of 25 kg/m². Follow-up time was defined as the interval between the final preoperative evaluation and the one-year postoperative weight measurement.

Laboratory parameters were obtained from the institutional electronic medical records and included the following tests: 25-hydroxyvitamin D, folic acid, total calcium, ionized calcium, serum iron, ferritin, hemoglobin, hematocrit, albumin, total proteins, parathyroid hormone (PTH), total iron-binding capacity, transferrin, and vitamin B12. Cutoff values were defined according to published bariatric guidelines^14^, and when a specific cutoff was unavailable, the hospital’s institutional laboratory reference values were used. Preoperative laboratory values corresponded to the last tests performed before surgery, while postoperative values corresponded to results obtained approximately 12 months after surgery.

Dietary intake was assessed using a 24-hour dietary recall (24HR) conducted by a registered dietitian during the one-year postoperative consultation. Data were analyzed using WebDiet software to estimate total energy, proteins, carbohydrates, lipids, and dietary fiber daily intake. Protein adequacy was evaluated based on the minimum threshold of 60 g/day, as recommended by current bariatric nutritional guidelines^12^ ^,^ ^13^. Dietary fiber intake was compared against the recommendations established for the general adult population^27^ ^,^ ^28^, as no specific fiber intake targets for bariatric surgery patients have been established in current nutritional guidelines.

Food tolerance was evaluated using the questionnaire proposed by Suter et al.^15^ and adapted to Portuguese by Godoy et al.^16^. The instrument assesses overall satisfaction with alimentation, meal timing throughout the day, tolerance to eight food groups, and the frequency of vomiting/regurgitation. A final score is derived from sections 1, 3, and 4 of the questionnaire and ranges from 1 to 27, with higher scores indicating better food tolerance.

Gastrointestinal symptoms were assessed using the Gastrointestinal Symptom Rating Scale (GSRS), originally developed by Svedlund et al.^17^ and later validated in Portuguese by Souza et al.^18^. This questionnaire consists of 15 items grouped into five domains: diarrhea, constipation, abdominal pain, reflux, and indigestion. Each item is rated on a seven-point Likert scale, where 1 indicates the absence of symptoms and 7 the highest frequency or intensity of symptoms. Domain scores were calculated according to the original instrument guidelines, with higher scores indicating greater symptom severity.

The questionnaires were administered during the outpatient follow-up visit approximately one year after surgery. Follow-up time was defined as the interval between the date of surgery and questionnaire administration.

Statistical analyses were performed using R software (version 4.5.5), adopting a significant level of 5%. Categorical variables were expressed as counts and percentages, while continuous variables were expressed as mean ± standard deviation (SD). Gastrointestinal symptoms and food tolerance were assessed by questionnaire scores, which were calculated according to the original validation guideline and estimated through linear regression models adjusted for sex and age. Anthropometric and laboratory outcomes (analyzed as continuous variables) were also estimated through linear regression models and additionally adjusted for their respective preoperative baseline values.

## RESULTS

Baseline characteristics are presented in Table 1. The study included 46 patients (30 RYGB vs 16 OAGB), predominantly female (85%), with a mean age of 50 ± 9 years. OAGB patients had significantly higher preoperative weights and BMIs when compared to RYGB patients (*P*<0.001), reflecting the institutional protocol for OAGB indication, which required a preoperative BMI>50kg/m^2^. Sex distribution also differed, with a higher proportion of males in the OAGB group (*P*=0.04). No significant differences were observed for age, height, or follow-up duration.

**TABLE 1:** Baseline characteristics.

Characteristic	Overall (n = 46)	RYGB (n = 30)	OAGB (n = 16)	***P*-value**
Female sex, n (%)	39 (85%)	28 (93%)	11 (69%)	0.04
Age (years)	50 (± 9)	49 (± 10)	51 (± 6)	0.3
Height (m)	1.62 (± 0.10)	1.61 (± 0.09)	1.65 (± 0.10)	0.2
Preoperative weight (kg)	125 (± 20)	115 (± 12)	144 (± 18)	<0.001
Preoperative BMI (kg/m²)	47.5 (± 5.3)	44.6 (± 3.6)	52.9 (± 3.3)	<0.001
Maximum preoperative weight (kg)	137 (± 23)	126 (± 16)	156 (± 22)	<0.001
Maximum preoperative BMI (kg/m²)	51.9 (± 6.1)	49.0 (± 4.5)	57.3 (± 4.9)	<0.001

Mean (SD) for continuous variables; n (%) for categorical variables. BMI = body mass index. Welch t-test or Fisher’s exact test.

After adjustment for preoperative BMI, age, and sex, no significant differences were found between groups in one-year postoperative weight or BMI. Both procedures achieved comparable weight loss outcomes one year after surgery, whether assessed by TWL and EWL, and regardless of whether it was considered the last preoperative or maximum preoperative weight (all *P*>0.05). The mean follow-up time between surgery and postoperative weight measurement was 12 ± 3 months overall (RYGB 13 ± 2 vs OAGB 12 ± 2 months; *P*=0.2). Anthropometric outcomes comparison are presented in Table 2 and Figure 1.

**TABLE 2:** Anthropometric outcomes one year after surgery.

	RYGB (n=30) Mean (95%CI)	OAGB (n=16) Mean (95%CI)	Mean difference OAGB vs RYGB (95% CI)	***P*-value**
Postoperative weight (kg)	89.27 (84.43-94.12)	93.17 (85.40-100.94)	3.90 (−6.94 to 14.74)	0.47
Postoperative BMI (kg/m²)	33.87 (32.28-35.45)	35.39 (32.58-38.20)	1.53 (−2.23 to 5.29)	0.42
**Based on the last preoperative weight**				
TWL (%)	29.55 (25.98-33.11)	26.32 (20.70-31.94)	−3.23 (−11.10 to 4.65)	0.41
EWL (%)	63.36 (55.59-71.13)	58.29 (46.04-70.54)	−5.07 (−22.23 to 12.09)	0.55
**Based on the maximum preoperative weight**				
TWL (%)	34.71 (30.96-38.46)	33.56 (27.65-39.47)	−1.15 (−9.43 to 7.13)	0.78
EWL (%)	68.20 (61.63-74.78)	66.05 (55.68-76.41)	−2.16 (−16.68 to 12.36)	0.77

Adjustment means (95%CI) from linear regression models adjusted for sex, age, and preoperative BMI. BMI = body mass index. TWL = total weight loss. EWL = excess weight loss.

**FIGURE 1:**
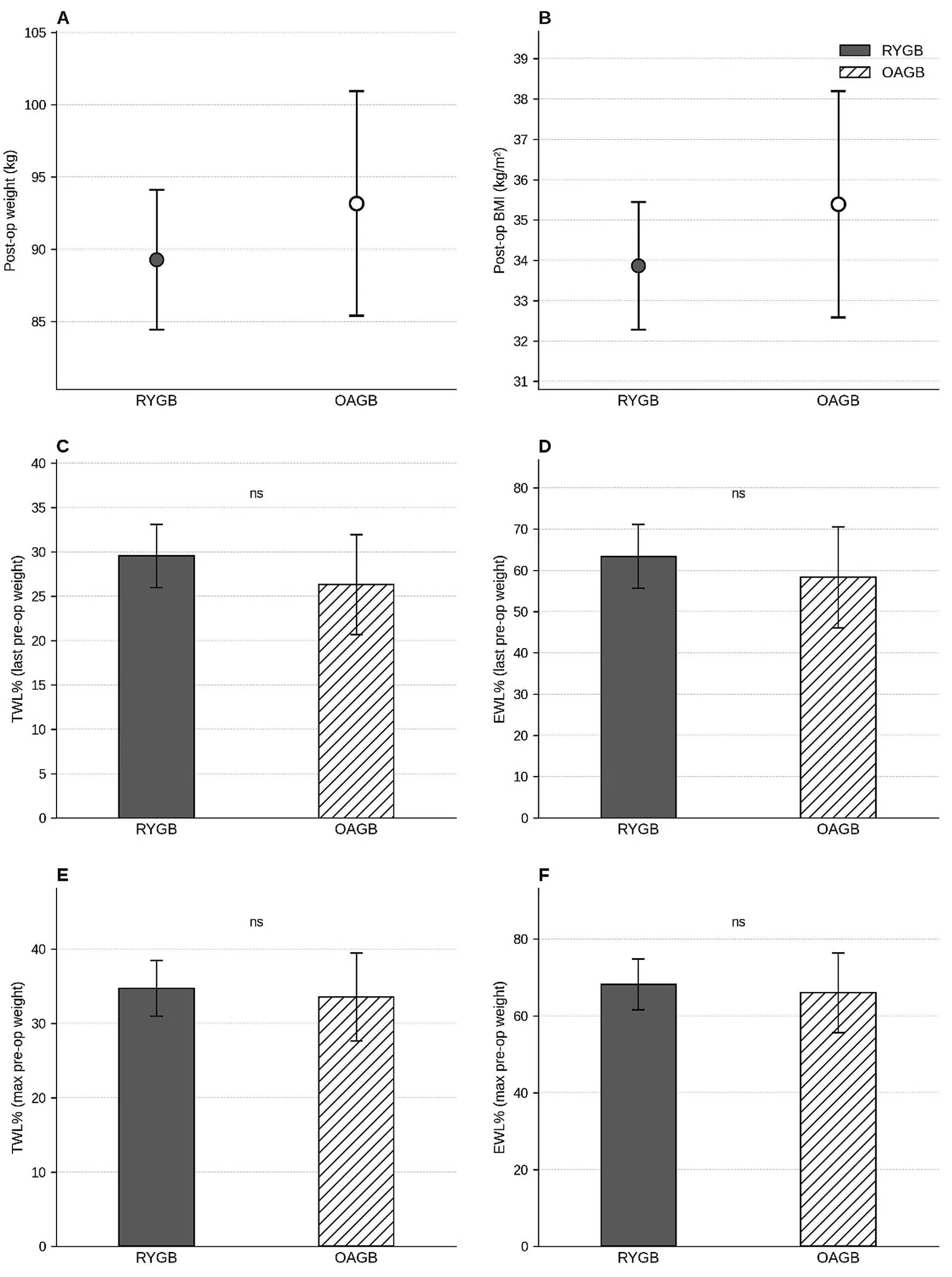
Anthropometric outcomes one year after surgery. (A) Postoperative weight; (B) postoperative BMI; (C) TWL based on last preoperative weight; (D) EWL based on last preoperative weight; (E) TWL based on maximum preoperative weight; (F) EWL based on maximum preoperative weight. Values represent adjusted means and 95%CI from linear regression models adjusted for sex, age, and preoperative BMI. BMI = body mass index. TWL = total weight loss. EWL = excess weight loss. ns = not significant.

Mean macronutrient intake, as well as total energy intake, was similar between groups, with no statistically significant differences observed between the surgical techniques (Table 3). Mean protein intake met the minimum recommended threshold of 60 g/day for bariatric patients in both groups. Fiber intake was below the general population recommendation of 25 g/day in both procedures. The mean interval between surgery and 24HR application was 18 ± 7 months overall (RYGB 17 ± 7 vs OAGB 19 ± 8 months; *P*=0.3).

**TABLE 3:** Dietary Intake one year after surgery.

Variable	Overall (N=46)	RYGB (N=30)	OAGB (N=16)	p-value
Protein (g/day)	62 (22)	60 (21)	67 (23)	0.3
Lipid (g/day)	38 (19)	41 (22)	34 (11)	0.2
Carbohydrate (g/day)	145 (59)	150 (65)	138 (45)	0.5
Fiber (g/day)	12.6 (7.1)	13.6 (7.6)	10.8 (5.9)	0.2
Energy (kcal/day)	1136 (388)	1184 (403)	1044 (353)	0.2

Mean (SD); Welch’s t-test.

After adjustment for preoperative values, no statistically significant between-group differences were found in any postoperative laboratory parameter, with exception of ionized calcium (mean difference: 0.08 mg/dL; 95%CI 0.01-0.15; *P*=0.03); however, both groups remained within the normal clinical range, suggesting limited clinical relevance. The most prevalent finding in both groups was 25-hydroxyvitamin D deficiency (≤30 ng/mL), affecting 82% of RYGB and 92% of OAGB patients. Elevated PTH (≥65 pg/mL) was observed in 50% of RYGB and 75% of OAGB patients. 

Although OAGB patients showed numerically lower serum iron (75 vs 92 µg/dL) and transferrin saturation (28% vs 38%), these differences did not reach statistical significance after adjustment (*P*=0.93 and *P*=0.62, respectively). No cases of anemia were identified. Vitamin B12, folic acid, albumin, and total protein were adequate in both groups with no significant between-group differences. The mean interval between surgery and post-operative laboratory collection was 12 ± 3 months in both groups (*P*=0.2). Laboratory parameters are presented in Table 4.

**TABLE 4:** Laboratory parameters at one-year post-surgery.

Variable	Overall N = 46	RYGB N = 30	OAGB N = 16	Mean difference OAGB vs RYGB (95%CI)	***P*-value**
25-OH Vitamin D (ng/mL)	30 (10)	30 (10)	29 (11)	0.06 (−7.37 to 7.48)	0.99
Deficiency ≤30 ng/mL, n (%)	30 (86%)	18 (82%)	12 (92%)	-	-
PTH (pg/mL)	86 (28)	78 (18)	101 (40)	7.11 (−5.93 to 20.15)	0.27
Elevated ≥65 pg/mL, n (%)	19 (59%)	10 (50%)	9 (75%)	-	-
Folic acid (ng/mL)	14.2 (4.4)	14.4 (4.4)	14.0 (4.6)	−1.45 (−4.26 to 1.35)	0.30
Deficiency <3.1 ng/mL, n (%)	0 (0%)	0 (0%)	0 (0%)	-	-
Vitamin B12 (pg/mL)	535 (310)	496 (279)	614 (362)	83.37 (−95.16 to 261.90)	0.35
Deficiency <200 pg/mL, n (%)	2 (5.1%)	2 (8.0%)	0 (0%)	-	-
Total calcium (mg/dL)	9.10 (0.36)	9.08 (0.37)	9.15 (0.36)	0.05 (−0.21 to 0.31)	0.70
Deficiency <9 mg/dL, n (%)	6 (17%)	3 (14%)	3 (23%)	-	-
Ionized calcium (mg/dL)	4.87 (0.13)	4.84 (0.13)	4.94 (0.10)	0.08 (0.01 to 0.15)	0.03*
Deficiency <4.5 mg/dL, n (%)	0 (0%)	0 (0%)	0 (0%)	-	-
Serum iron (µg/dL)	86 (32)	92 (32)	75 (30)	−0.98 (−22.47 to 20.51)	0.93
Deficiency ≤50 µg/dL, n (%)	3 (7.9%)	1 (4.2%)	2 (14%)	-	-
Ferritin (ng/mL)	221 (225)	219 (194)	226 (286)	29.25 (−83.67 to 142.18)	0.60
Deficiency <12 ng/mL, n (%)	0 (0%)	0 (0%)	0 (0%)	-	-
Transferrin saturation (%)	35 (17)	38 (18)	28 (11)	−2.36 (−12.09 to 7.37)	0.62
Deficiency <20%, n (%)	5 (16%)	3 (15%)	2 (17%)	-	-
Transferrin (µg/dL)	247 (38)	251 (38)	237 (38)	−10.48 (−32.84 to 11.88)	0.35
Deficiency <200 µg/dL, n (%)	2 (5.9%)	1 (4.8%)	1 (7.7%)	-	-
TIBC (µg/dL)	280 (38)	284 (38)	271 (36)	−21.44 (−43.13 to 0.26)	0.053
Elevated ≥450 µg/dL, n (%)	0 (0%)	0 (0%)	0 (0%)	-	-
Hemoglobin (g/dL)	12.72 (1.13)	12.75 (0.96)	12.65 (1.45)	−0.18 (−0.74 to 0.39)	0.53
Deficiency <12 g/dL, n (%)	0 (0%)	0 (0%)	0 (0%)	-	-
Hematocrit (%)	38.4 (3.3)	38.4 (2.8)	38.3 (4.4)	−0.13 (−1.87 to 1.62)	0.88
Deficiency <35%, n (%)	1 (2.3%)	1 (3.6%)	0 (0%)	-	-
Albumin (g/dL)	4.29 (0.30)	4.33 (0.34)	4.21 (0.20)	−0.11 (−0.31 to 0.10)	0.30
Deficiency <3.5 g/dL, n (%)	1 (2.3%)	1 (3.7%)	0 (0%)	-	-
Total protein (g/dL)	6.93 (0.34)	6.96 (0.36)	6.88 (0.29)	−0.10 (−0.32 to 0.13)	0.40
Deficiency <6.4 g/dL, n (%)	6 (14%)	4 (15%)	2 (13%)	-	-

Mean (SD) for continuous variables; n (%) for categorical variables. *P*-values from linear regression models adjusted for pre-operative values of each parameter. PTH = parathyroid hormone. TIBC = total iron-binding capacity. *P<0.05.

Mean (SD) for continuous variables; n (%) for categorical variables. *P*-values from linear regression models adjusted for pre-operative values of each parameter. PTH = parathyroid hormone; TIBC = total iron-binding capacity; *P<0.05.

Gastrointestinal symptoms and food tolerance outcomes are presented in Figure 2. OAGB patients presented significantly lower GSRS scores for diarrhea (mean difference: −1.35; 95%CI −2.59 to −0.10; *P*=0.03), indigestion (mean difference: −1.19; 95%CI −1.94 to −0.43; *P*=0.003), and total score (mean difference: −0.85; 95%CI −1.46 to −0.24; *P*=0.007) compared to RYGB. No significant differences were observed for reflux, abdominal pain, or constipation domains.

**FIGURE 2:**
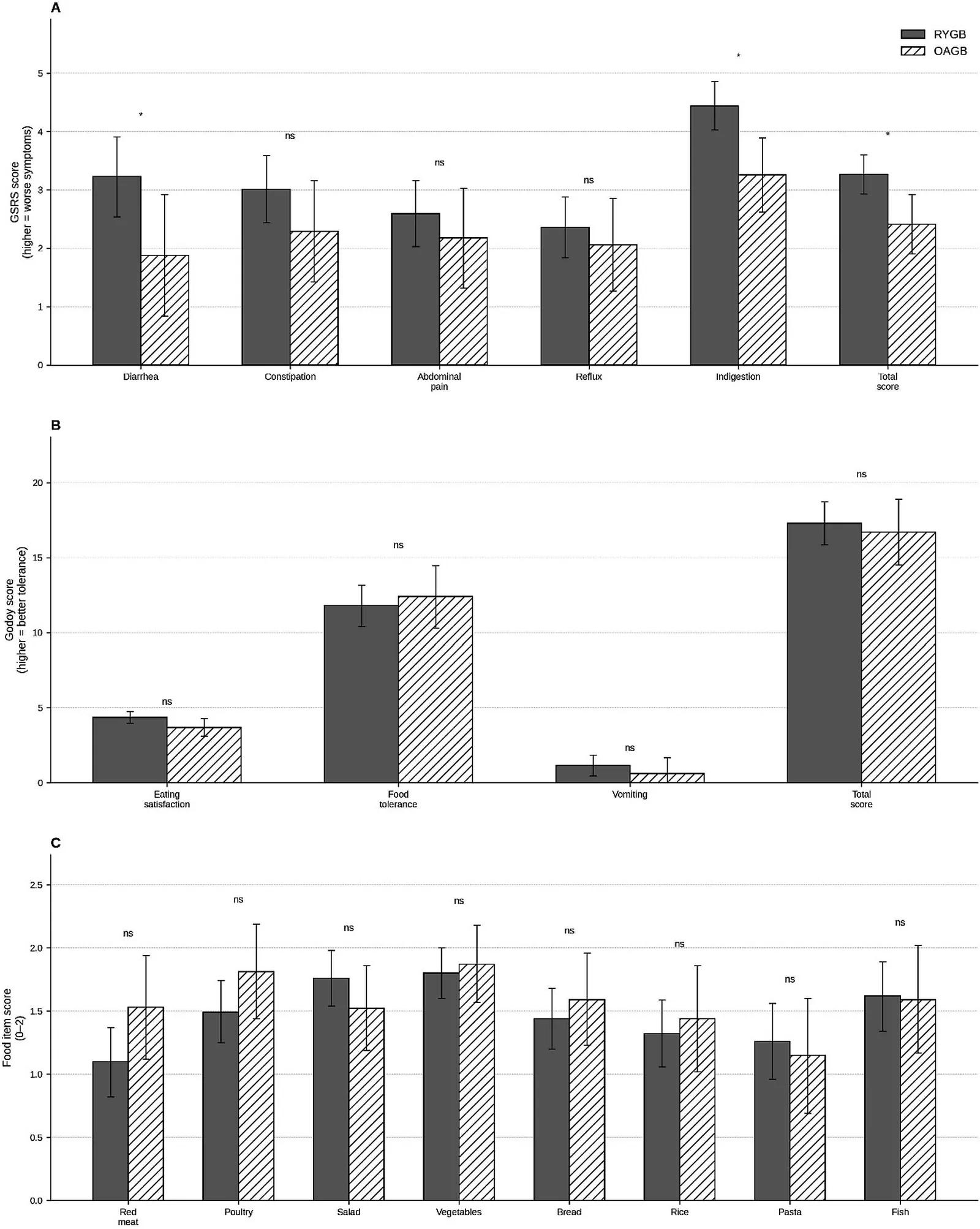
Gastrointestinal symptoms and food tolerance. (A) GSRS and total score (higher scores indicate worse gastrointestinal symptoms); (B) Godoy questionnaire and total score (higher scores indicate better food tolerance); (C) individual food item tolerance from Godoy’s questionnaire. Values represent the adjusted means and 95%CI from linear regression models adjusted for age and sex. *P<0.05; ns = not significant.

Food tolerance assessed by the Godoy questionnaire was comparable between groups, with no statistically significant differences in total score or any individual domain, including eating satisfaction, tolerance to the eight food groups, and the frequency of vomiting/regurgitation. When asked about intolerances beyond the items covered by the Godoy questionnaire, RYGB patients most frequently reported intolerance to dairy products (23.3%) and high-sugar foods (20.0%), followed by fruits (10.0%), legumes, leafy vegetables, and fatty foods (6.7% each). In the OAGB group, dairy products and high-sugar foods were also the most frequently reported intolerances (18.8% each), followed by fruits, legumes, and eggs (6.3% each). No statistically significant between-group differences were found for any food category (Fisher’s exact test, all *P*≥0.05). The mean interval between surgery and questionnaire administration was 18 ± 7 months overall (RYGB 17 ± 7 vs OAGB 19 ± 8 months; *P*=0.3).

## DISCUSSION

The present study compared food tolerance, gastrointestinal symptoms, and nutritional status one year after OAGB or RYGB. As expected from the institutional inclusion criteria (which required a preoperative BMI >50 kg/m^2^ for OAGB), baseline weight and BMI were significantly higher among OAGB patients, and the OAGB group had a higher proportion of males. These imbalances were addressed through linear regression models adjusted for sex, age, and preoperative BMI.

After adjustment for confounders, no significant differences were found in TWL or EWL at one year between groups, whether based on the last or maximum preoperative weight. These findings are consistent with the literature. Karagul et al.^19^, in a randomized prospective trial conducted in Turkey including 38 patients (OAGB: n = 20; RYGB: n = 18; mean preoperative BMI of ~45 and ~47 kg/m², respectively), reported comparable TWL between techniques at one year (OAGB: 36.6%; RYGB: 34.1%; *P*=0.282). Furthermore, a systematic review and meta-analysis by Musella et al.^20^, including 22 studies and 14,692 patients, demonstrated that OAGB can present wide variation in long-term weight loss outcomes, with %TWL ranging from 24.6% to 48.8% and %EWL ranging from 56.3% to 94.0% over five years of follow-up.

With respect to laboratory parameters, vitamin D deficiency was highly prevalent in both groups (RYGB 82% vs OAGB 92%), and elevated PTH levels were more frequent in OAGB patients than RYGB patients (75% vs 50%, respectively), with no significant between-group difference after adjustment for baseline values. The high prevalence of vitamin D deficiency after 12 months of OAGB has been reported previously^21^ ^,^ ^22^. Barzin et al.^21^, in a prospective cohort study in Tehran Obesity Treatment Center, Iran, including 193 OAGB patients with a biliopancreatic limb of 160 cm, reported a secondary hyperparathyroidism prevalence of 17.1% at one year, substantially lower than the 75% observed in our OAGB group. The differences may be partly related to differences in biliopancreatic limb length between protocols. Luger et al.^22^, in a randomized controlled trial conducted in Vienna, Austria, including 50 OAGB individuals, reported that bone metabolism biomarkers and secondary hyperparathyroidism rates were influenced by postoperative vitamin D supplementation adequacy, ranging from 10 to 24% prevalence depending on the supplementation group.

Although secondary hyperparathyroidism has been widely described after RYGB, our findings suggest that OAGB patients are similarly susceptible^23^ ^,^ ^24^. The only significant difference observed was in ionized calcium; notwithstanding, it was of small magnitude and remained within normal range, likely reflecting a type I error given the limited sample size.

It is noteworthy that our sample rates of vitamin D deficiency and secondary hyperparathyroidism remained high in both groups, despite regular supplementation. One contributing factor may be the exclusive use of calcium carbonate rather than calcium citrate in our institution, due to its limited availability in the Brazilian public health system, despite bariatric nutritional guidelines recommending calcium citrate as the preferred formulation in the postoperative period^12^ ^,^ ^13^.

Regarding iron metabolism, although no patient developed anemia, OAGB patients showed numerically lower serum iron and transferrin saturation, consistent with prior reports of reduced iron markers after OAGB, even under supplementation, likely due to the bypass of the duodenum and proximal jejunum (the main sites of iron absorption). A meta-analysis by Kermansaravi et al.^25^, including 26 studies with 11,015 patients, reported an overall 16% prevalence of iron-deficiency anemia (IDA) after OAGB, with variation in IDA prevalence according to biliopancreatic limb length. However, the authors reported an important limitation regarding the IDA definition criteria among the included studies. It is worth mentioning that our OAGB protocol used a biliopancreatic limb length of 200 cm from the ligament of Treitz, corresponding to the ≥200 cm subgroup in the meta-analysis, which had a reported IDA prevalence of 9%^25^. In our cohort, no patient developed established anemia, although subclinical alterations in iron metabolism were observed and may represent early stages preceding IDA. Vitamin B12, folic acid, albumin, and total protein were within normal limits in both groups, suggesting adequate adherence to supplementation under structured follow-up.

Dietary intake analysis was also similar between groups. Mean protein consumption met the minimum recommended threshold of 60 g/day in both techniques. Nevertheless, recent evidence suggests that absolute fixed protein threshold may not fully address individual demands, with recommendations based on g/kg of adjusted body weight being considered more appropriate to meet individual needs^26^. Despite these considerations, the adequate laboratory markers of protein status observed in this study suggest that the reported intake was sufficient to maintain protein balance during the postoperative period.

Fiber intake was below the general adult population recommendations^27^ ^,^ ^28^ in both groups (RYGB 13.6 g/day vs OAGB 10.8 g/day), though this was not accompanied by reported intolerance to fiber-rich foods according to the food tolerance questionnaire, suggesting that reduced fiber consumption reflects dietary choices rather than food intolerance. No significant differences were found in carbohydrate, lipid, or energy intake, with respective intake consistent with the expected hypocaloric pattern of the first postoperative year.

It is noteworthy that the use of a single 24HR has limitations regarding accuracy, since it is a memory-based instrument and may not precisely represent the patient’s habitual diet. However, it may still provide useful information about the eating pattern and dietary habits. This method was chosen because it is routinely applied in our outpatient nutritional follow-up protocol and reflects the practical conditions of care provided by our service.

The evaluation of gastrointestinal symptoms using the GSRS revealed a favorable profile for OAGB, with significantly lower scores for diarrhea, indigestion, and total score compared to RYGB. These findings contrast with previous reports in the literature. Zarshenas et al. described greater frequency of diarrhea, steatorrhea, and reflux in OAGB patients compared to RYGB, particularly in the early postoperative period^29^. The YOMEGA trial similarly reported higher diarrhea rates in OAGB at both three months (OAGB 26% vs RYGB 3.2%) and two years (OAGB 19.7% vs RYGB 7%)^30^. Additionally, a multicenter study across Israel and Portugal reported a high frequency of gastrointestinal side effects after OAGB, including diarrhea, nausea, and abdominal pain, with symptoms tending to persist over time^31^. The divergence between our results and these studies may reflect differences in follow-up duration, patient selection, biliopancreatic limb length, and the structured multidisciplinary nutritional counseling provided in our service.

Regarding reflux, although biliary reflux has been a recurrent concern after OAGB, our patients showed low reflux scores on the GSRS. These findings are consistent with Robert et al.^30^, who also relied on self-reported methods and similarly found low reflux rates. While questionnaire-based assessment has inherent limitations in accurately characterizing reflux, these results suggest that, under structured multidisciplinary follow-up, reflux symptoms may not impact the quality of life of OAGB patients.

Food tolerance assessed through the Godoy questionnaire did not differ significantly between surgical techniques, with comparable scores for eating satisfaction, food tolerance, and frequency of vomiting/regurgitation. These findings align with Kessler et al.^32^, who reported high food tolerance scores and eating satisfaction in OAGB patients at 12-20 months, despite the presence of mild-to-moderate gastrointestinal effects. As in our study, the authors noted that gastrointestinal symptoms and food tolerance appear to be dissociated outcomes; patients may experience digestive symptoms without a corresponding reduction in food tolerance. 

Dairy products and high-sugar foods were the most frequently reported intolerances in both groups, a pattern likely related to dumping syndrome, a well-known complication after bariatric procedures that was not formally assessed in this study. Notably, intolerance to fiber-rich foods was not frequently reported in either group, suggesting that the low mean fiber intake observed reflects dietary choices rather than food tolerance limitations.

Some limitations in this study should be considered. First, the relatively small sample size and the single-center design may limit the statistical power to detect subtle differences between surgical techniques. Second, the retrospective design restricts control over potential confounding variables and relies on the accuracy of electronic medical records. Third, baseline differences between groups, especially regarding preoperative weight and BMI, were mitigated through statistical adjustment, but residual confounding cannot be excluded. Additionally, individual sociodemographic variables, such as income and educational level, were not collected in the present study and should therefore be acknowledged as a limitation, as socioeconomic factors may influence postoperative outcomes, including access to supplementation, adherence to follow-up, and lifestyle habits. Furthermore, dietary intake, gastrointestinal symptoms, and food tolerance were assessed using self-reported instruments, which are subject to recall bias. Finally, the evaluation of reflux was limited to questionnaire-based methods; objective diagnostic tools (including upper endoscopy, pH monitoring, and manometry^33^ ^,^ ^34^) are planned as part of the broader institutional protocol to which this study belongs.

## CONCLUSION

Under structured multidisciplinary follow-up, OAGB and RYGB demonstrated comparable outcomes one year after surgery in terms of weight loss, dietary intake, and food tolerance. OAGB patients presented lower gastrointestinal symptom scores for diarrhea, indigestion, and total GSRS scores. High prevalence of vitamin D deficiency and subclinical iron metabolism alterations were observed in both groups, particularly following OAGB. These findings suggest that OAGB is a nutritionally safe alternative to RYGB in the medium term, but requires continuous nutritional monitoring, particularly regarding iron and vitamin D status.

## Data Availability

data-available-upon-request
